# Genetic Dissection and Breeding Potential of Carotenoid Content in Foxtail Millet (*Setaria italica*) Using a Major Gene Plus Polygene Model

**DOI:** 10.3390/plants15030486

**Published:** 2026-02-04

**Authors:** Rui Huang, Haigang Wang, Yimin Pan, Yongxiang Xie, Hui Zhi, Xia Liu, Yanzhen Wang, Xianmin Diao, Juanling Wang

**Affiliations:** 1 College of Agriculture, Shanxi Agricultural University, Taigu 030801, China; huangrui@sxau.edu.cn (R.H.); 18536494141@163.com (Y.P.); 202420155@stu.sxau.edu.cn (Y.X.); 2 Center for Agricultural Genetic Resources Research, Shanxi Agricultural University, Taiyuan 030031, China; wanghg@sxau.edu.cn (H.W.); liuxia1214lx@163.com (X.L.); wangyanzhen9605@163.com (Y.W.); 3 Key Laboratory of Sustainable Dryland Agriculture of Shanxi Province, Taiyuan 030031, China; 4 Institute of Crop Sciences, Chinese Academy of Agricultural Sciences, Beijing 100081, China; zhihui@caas.cn

**Keywords:** foxtail millet, carotenoid, nutritional breeding, genetic analysis

## Abstract

Carotenoid content is a key trait that defines the unique characteristics of foxtail millet varieties. Varieties with different levels of carotenoids often show distinct genetic features and nutritional profiles. However, the genetic basis of carotenoid content in foxtail millet remains mostly unknown. In this study, we explored the genetic basis of carotenoid content using a recombinant inbred line (RIL) population of 305 lines derived from two parental accessions, JG21 (high-carotenoid, 16.75 mg·kg^−1^) and JG25 (low-carotenoid, 0.93 mg·kg^−1^). The results showed that the RIL population exhibited continuous phenotypic variation and significant transgressive segregation for carotenoid components (lutein, zeaxanthin, β-cryptoxanthin) and kernel color (measured by b* value), with zeaxanthin reaching 8.47 mg·kg^−1^, significantly surpassing the higher parent (3.44 mg·kg^−1^) in 24DY. To ensure that enhancing this nutritional trait does not compromise grain yield, we analyzed its relationship with key agronomic traits, testing for pleiotropic trade-offs. Notably, carotenoid content showed no significant correlation with any of the 8 key agronomic traits (r ranged from −0.11 to 0.08, all *p* > 0.05), suggesting no apparent trade-off, although fine-mapping is needed to separate pleiotropy from tight linkage for concurrent improvement. Genetic modeling analysis revealed that carotenoid content is stably controlled by three major-gene pairs plus polygenes (MX3-AI-A model), with major-gene heritability of 96.65% and polygene heritability of 3.35%. Based on this framework, three elite RILs with >23% higher carotenoid and superior agronomic performance were identified and advanced to marker-assisted backcrossing. These results provide a clear genetic framework and immediate breeding resources for marker-assisted selection, enabling the development of high-yielding, carotenoid-enriched foxtail millet varieties without compromising agronomic value.

## 1. Introduction

Foxtail millet (*Setaria italica* (L.) Beauv.) is an ancient diploid C4 cereal crop originating from China. Renowned for its exceptional drought tolerance and adaptability to infertile soils, it is a staple food in the arid and semi-arid regions of Northern China [1,2]. Beyond its resilience, foxtail millet grain possesses outstanding nutritional value after dehulling, being rich in proteins, fatty acids, vitamins, and carotenoids [3,4]. Therefore, carotenoid biofortification through breeding has emerged as a sustainable strategy for enhancing nutritional security worldwide. However, the efficient breeding of high-carotenoid varieties is hampered by an incomplete understanding of their genetic inheritance patterns in foxtail millet.

Kernel color, a critical indicator of commercial quality and a primary factor influencing consumer preference, has been established to have a highly significant positive correlation with carotenoid content [5,6,7]. Carotenoids are the primary pigments responsible for the yellow coloration of foxtail millet grains [8]. The predominant carotenoids found in foxtail millet include lutein and zeaxanthin, along with trace amounts of β-carotene and cryptoxanthin [8]. These pigments belong to the tetraterpenoid class and are widely distributed throughout nature. They serve as fundamental components of the photosynthetic apparatus by participating in photosystem assembly and facilitating light-harvesting processes [9,10]. Beyond their role as pigments, certain carotenoids serve as essential precursors for vitamin A synthesis, which is crucial for human health [11]. However, as essential micronutrients that cannot be synthesized by humans, carotenoids must be obtained from plant-derived foods [12,13].

The major gene plus polygene mixed inheritance model provides an efficient framework for dissecting complex quantitative traits by simultaneously estimating major gene effects (additive/dominance) and polygenic background contributions. This phenotypic-based approach is particularly valuable for early-generation selection in breeding programs where genomic resources are limited, as it does not require prior molecular marker information—unlike QTL mapping or GWAS. This model has been successfully applied to various traits in different crops, and the genetic architecture of carotenoid accumulation has been extensively investigated in several major cereals, revealing diverse inheritance patterns. In maize, studies suggest a predominant role of non-additive gene effects [14], while QTLs under both additive and non-additive gene actions have also been identified [15]. Similarly, studies in wheat indicate that carotenoid content is controlled by both major genes and polygenes, with additive effects playing a significant role [16,17]. However, the genetic dissection of carotenoid content in foxtail millet, a model C4 plant and nutritionally dense crop, remains methodologically fragmented. Decades of visual selection for kernel yellowness have achieved only slow and inconsistent gains, underscoring the need for a quantitative, heritability-driven framework to accelerate carotenoid biofortification. Li et al. [18] successfully applied the major gene plus polygene model to dissect agronomic traits, but never extended this robust framework to quality traits like carotenoids. This leaves a critical gap in nutritional breeding. Gou et al. [19] mapped QTLs for kernel color (b* value), yet the b value is merely an indirect proxy. It integrates carotenoid content with light scattering effects from color, making it impossible to disentangle the genetic architecture of specific components like lutein and zeaxanthin. Nor can it estimate heritability for marker-assisted selection. This disconnect—where mature models exist for agronomic traits but remain absent for nutritional traits—obscures breeding relevant gene actions and precludes precise genetic improvement of carotenoid content [20].

A critical biological concern in biofortification is the potential trade-off between nutritional quality and grain yield, as resource allocation may shift toward secondary metabolites at the expense of biomass [20]. In foxtail millet, studies have shown that grain color (b* value) and carotenoid content exhibit significant positive correlations with appearance quality and palatability [21,22], highlighting the importance of these traits for consumer preference. However, the relationship between carotenoid accumulation and yield-related traits can be complex, with studies in cereals reporting varied pleiotropic effects [14,16]. Therefore, explicitly testing for genetic independence between carotenoid and agronomic traits is essential to justify concurrent improvement strategies.

To address this knowledge gap, we developed a recombinant inbred line (RIL) population from a cross between the low-carotenoid cultivar Jiugu 25 and the high-carotenoid cultivar Jingu 21. While this single-cross RIL population provides a foundational framework for dissecting carotenoid inheritance, we acknowledge that conclusions require validation in multi-parent crosses to enhance generality. Still, the extreme phenotypic divergence between parents (18-fold) provides strong genetic variance and detection power for initial mapping and breeding proof of concept. The genetic architecture of carotenoid content was dissected through phenotypic evaluation across multiple environments. This investigation was guided by testing the following key hypotheses: (i) that carotenoid content is genetically independent from key agronomic traits; (ii) that it is controlled by a mixed major-gene plus polygene inheritance model; and (iii) that elite lines combining high carotenoid content with superior agronomic performance could be identified. The acceptance of these hypotheses was rigorously evaluated using the phenotypic correlation analyses, Akaike information criterion (AIC), and χ^2^ tests for genetic model fitting.

## 2. Results

### 2.1. Variation in Carotenoid Content and b* Value

**Figure 1:**
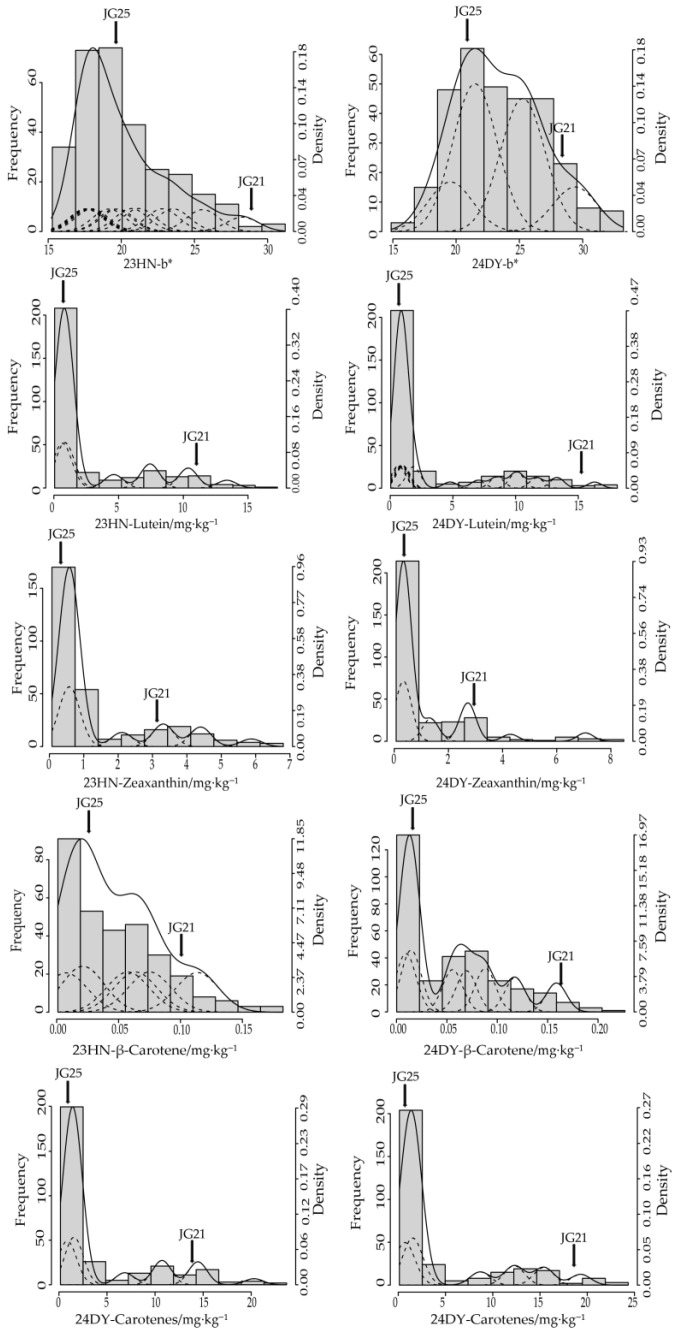
Frequency (column), fitted mixed (solid line) and its component (dotted line) distributions of all traits in the RIL population under two environments.

**Figure A1:**
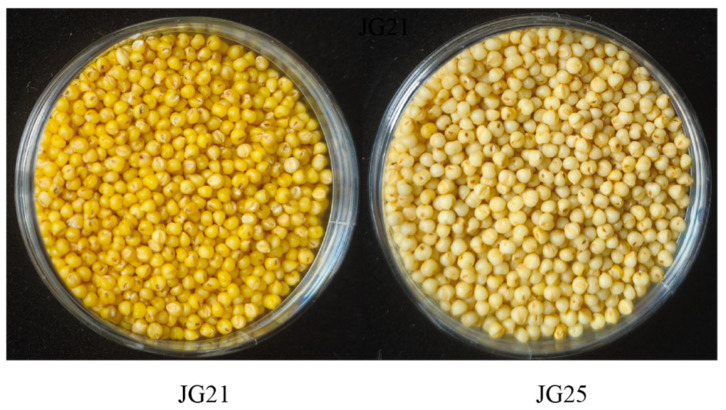
Representative images of kernel color for the two parents.

Phenotypic analysis revealed a clear contrast between parents JG21 (high carotenoid, yellow kernel) and JG25 (low carotenoid, white kernel), and the RIL mean for lutein, zeaxanthin, β-carotene, total carotenoid, and b* value were all intermediate between the two parents (Figure A1). In JG21, the total carotenoid content was 16.745 mg·kg^−1^, with average levels of lutein, zeaxanthin, and β-carotene at 13.16, 3.45, and 0.13, respectively. In contrast, the JG25 showed a significantly lower total content of only 0.93 mg·kg^−1^, with constituent levels of 0.57, 0.35, and 0.02. However, based on the phenotypic analysis of the RIL population and parental lines under two environments (23 HN and 24 DY), extensive transgressive segregation beyond the parental range was observed for all traits. For instance, zeaxanthin reached 8.47 mg·kg^−1^ in 24DY, significantly surpassing the higher parent (3.44 mg·kg^−1^), while the peak levels of lutein and total carotenoid reached 1.34 to 1.43 times that of the higher parent (Table 1). Coefficients of variation (CV) ranged from 78.96% to 137.04%, indicating high phenotypic variability. The frequency distributions for these traits were continuous and right-skewed, visually confirming the transgressive segregation (Figure 1). This significant variation and segregation make the population highly suitable for genetic inheritance analysis and selection studies.

### 2.2. Correlations Between Agronomic Traits and Carotenoid Accumulation

**Figure 2:**
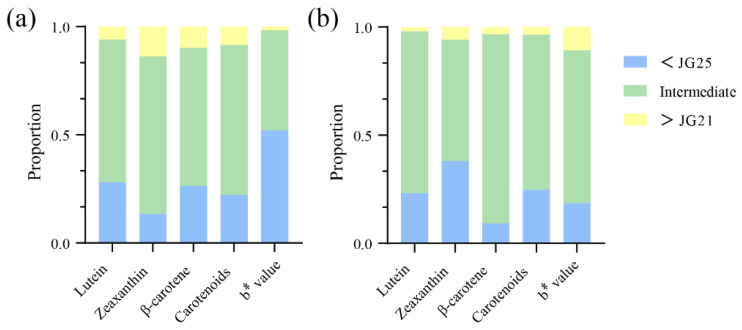
Stacked bar chart showing the proportion of transgressive families for carotenoid components and b value. Families are categorized as exceeding the high-parent (>JG21), falling below the low-parent (<JG25), or exhibiting intermediate values ((**a**): 23HN; (**b**): 24DY).

**Figure 3:**
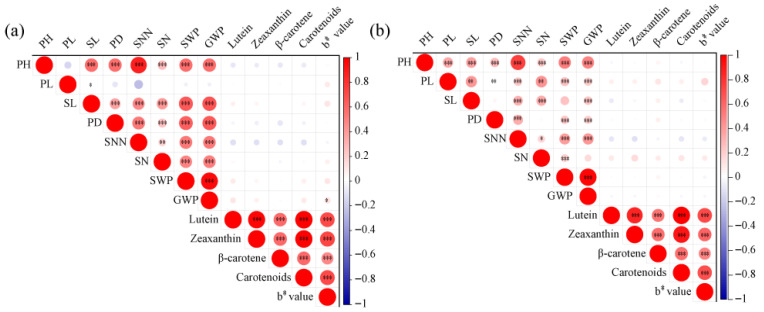
Correlation analysis of agronomic traits, carotenoid content, and b* value under different environments ((**a**): 23HN; (**b**): 24DY). Note: Circle size and color represent the strength and direction of the correlation. *: *p* < 0.05, **: *p* < 0.01, ***: *p* < 0.001.

To test Hypothesis (i) from the Introduction, we first examined extreme families’ agronomic performance (Table 2), followed by correlation analysis across all RILs. First, phenotypic analysis revealed a substantial number of transgressive families, particularly those with values below the white parent (<JG25) for multiple traits, indicating a higher proportion of families with lower carotenoid content than the white parent (Figure 2). In contrast, in the 23HN, 26 families exhibited higher total carotenoid content than the high-carotenoid parent JG21, while only 11 families surpassed JG21 in the 24DY environment. This disparity is likely attributable to genotype-by-environment interactions. The extensive transgressive segregation observed, with RILs exceeding both parental values, indicates complementary favorable alleles from the parents recombining at multiple loci. For downstream breeding, we selected transgressive lines based on three criteria: consistent carotenoid content surpassing the high-parent (JG21) across environments, stable agronomic performance without trade-offs, and absence of deleterious traits. These selected lines serve as valuable donors for marker-assisted breeding. Analysis of agronomic traits showed no significant differences among the extreme white families, extreme yellow families, and the population mean in either the 23HN or 24DY environments (Table 2). Further correlation analysis indicated that, whereas the b* value was significantly and positively correlated with all carotenoid components (*p* < 0.01), most agronomic traits showed no significant association with any of them (Figure 3). Although no phenotypic trade-offs were detected at the level of trait correlation, further multi-environment yield trials combined with molecular dissection (e.g., fine-mapping) are required to confirm the absence of pleiotropic or linkage effects at the genetic level. Furthermore, the identification of stable transgressive families provides excellent parental candidates for direct use in breeding high carotenoid foxtail millet.

### 2.3. Genetic Model Selection and Parameter Estimation for Carotenoid Content and b* Value

Genetic models for carotenoid contents and b* value in the RIL population were fitted using the R package SEA across different environments to test our second hypothesis. According to the Akaike Information Criterion (AIC), the models with the smallest AIC value and the models with the second minimum AIC value close to the minimum were selected as candidate models (Table 3) [17]. All candidate models were tested for goodness of fit, including the Uniformity test (U_1_^2^, U_2_^2^, U_3_^2^), the Smirnov test (_n_W^2^), and the Kolmogorov test (D_n_), to determine the optimal model (Table A1). Taking the lutein content as an example, MX3-AI-A (AIC = 1225.443) and MX3-A-A (AIC = 1233.017) were selected as candidate models for 23HN. Likewise, for lutein in 24DY, 4MG-AI, and MX-3-AI-A were optimal models for the goodness of fit tests. The number of values below the statistical significance threshold for four models was 9, 3, 4, and 1, respectively. Combining the goodness-of-fit test results with the AIC values, we finally determined that MX3-AI-A (three additive–epistatic major gene pairs plus additive polygenes) was the optimal model for lutein in both environments.

Using the same method to determine the optimal genetic model for other traits, we observed an interesting pattern: similar to lutein, the best-fit model for zeaxanthin, β-carotene, and total carotenoids was also MX3-AI-A. For the b* value, the optimal model was MX2-IE-A (two major inhibitory gene pairs plus additive polygene) in 23HN, whereas 3MG-CEA (three major gene model with identical additive effects) best explained the data in 24DY. This environment-dependent genetic architecture suggests that b* value—while correlated with carotenoid content—may also be modulated by additional factors, such as non-carotenoid pigments or structural grain traits, whose expression is sensitive to environmental conditions.

First- and second-order genetic parameters for carotenoid content and b* value were estimated under optimal models in both environments (Table 4). With the exception of β-carotene in the 23HN, the major gene heritabilities of all carotenoid components exceeded 90%. For the b* value, the major gene heritability was 59.80% and the polygene heritability was 27.27% under the 23HN. In contrast, under the 24DY environment, the major gene heritability increased to 75.66%, with the environment accounting for 24.34% of the variation. In the 24DY, the additive effects of the three major genes controlling b* value were identical, each with a value of 1.7121. The parameter estimates of the optimal MX3-AI-A model indicate that all three pairs of major genes contribute substantially to carotenoid content, as evidenced by their significant additive effects (e.g., for total carotenoids in 24DY: da = 4.511, db = 2.827, dc = 3.610). Together with the high total major-gene heritability (h^2^_mg_ > 96%), this confirms that the trait is governed by the collective additive action of multiple major genes, consistent with the observed continuous phenotypic variation and right-skewed segregation in the RIL population (Figure 1).

### 2.4. Screening for Elite Families

**Figure 4:**
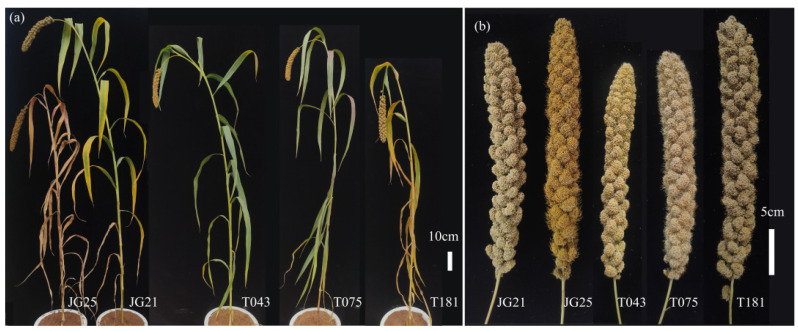
Phenotypic comparison of selected elite lines and the parental lines. Plant architecture (**a**) and panicles (**b**) of elite lines at maturity. Bar (**a**) = 10 cm; Bar (**b**) = 5 cm.

**Figure A2:**
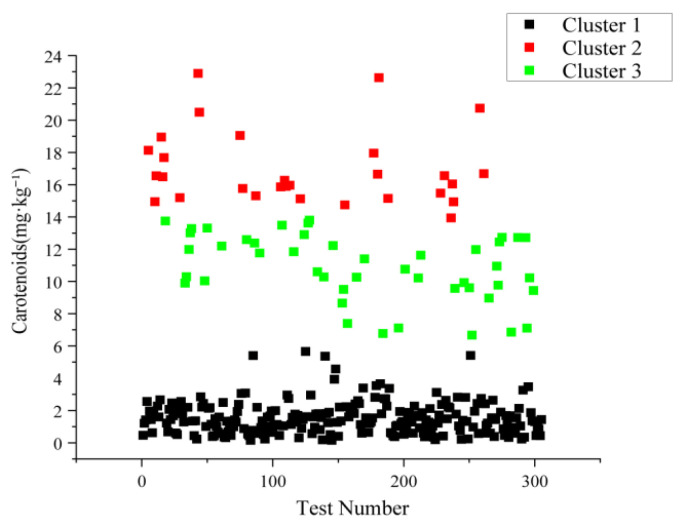
Cluster analysis of carotenoid content in the RIL population using the K-means method.

K-means clustering based on total carotenoid content partitioned the 305 lines into three groups comprising 230, 29, and 46 entries, respectively (Figure A2). Group I exhibited the lowest total carotenoid content (1.53 mg·kg^−1^), followed by Group III (10.85 mg·kg−1), and Group II possessed the highest content (16.97 mg·kg^−1^) (Table A2). By integrating carotenoid data with agronomic performance and heading date, we selected three yellow-kernel elite lines. T043 combined a relatively moderate plant height with a grain weight per panicle of 16.45 g; T075 was characterized by yellow kernels and early heading; T181 exhibited short plant height and an intense yellow kernel color (Figure 4). These elite lines maintained their high carotenoid content and agronomic performance across both 23HN and 24DY environments, indicating good environmental stability and providing a valuable genetic reservoir for breeding foxtail millet varieties with elevated carotenoid levels.

## 3. Discussion

### 3.1. Distribution of Kernel Color and Carotenoid Contents in the RIL Population

Kernel color serves as a crucial indicator of foxtail millet appearance quality, directly reflecting its overall quality. Studies demonstrate a highly significant positive correlation between kernel color, aroma, and palatability of cooked foxtail millet, with yellower kernels generally producing more fragrant porridge [21]. Consequently, kernel color not only acts as an immediate driver of consumer preference but also constitutes an important criterion for varietal quality evaluation [22]. Nonetheless, systematic investigations of foxtail millet kernel color remain limited. To investigate this trait, we constructed an RIL population from a cross between yellow and white foxtail millet varieties and performed a comprehensive genetic dissection of carotenoid accumulation and its relationship with agronomic traits. Previous studies have reported a wide spectrum of b* values in foxtail millet, from 12.14 to 75.35, across different genetic materials, including improved cultivars and specific breeding populations [5,22]. These reported ranges provide a critical context and validate the accuracy of our phenotyping protocol, as our measurements in the parental and RIL materials (b*: 14.24–33.91) fall within this established spectrum. Importantly, our study was not designed to merely report another range but to exploit a specifically created genetic resource—a biparental RIL population—to dissect the inheritance of this variation. The substantial variation in b* values across studies can be attributed to differences in the genetic backgrounds of the materials studied, variations in instrument settings and measurement protocols, and potentially to environmental factors influencing pigment expression. The yellow appearance of foxtail millet is intrinsically linked to the composition and concentration of its yellow pigments. Yang demonstrated a significant positive correlation between yellow pigment content and appearance quality using varieties exhibiting diverse kernel color [23]. Our results further corroborate this finding, showing a significant positive correlation between the b* value and total carotenoid content. Further investigation by Yang into yellow pigment content and specific carotenoid components revealed that lutein and zeaxanthin constitute 55–65% of the yellow pigments in foxtail millet grains, with reported ranges of 0.09–16.99 mg·kg^−1^ for lutein and 0.05–4.88 mg·kg^−1^ for zeaxanthin [6]. Similarly, Liu assessed lutein and zeaxanthin concentrations in 200 cultivated foxtail millet varieties from different provinces and found that they ranged from 2.216–3.474 mg·kg^−1^ and 7.308–9.989 mg·kg^−1^, respectively [24]. In this study, we found that lutein concentration ranged from 0.04 to 18.11 mg·kg^−1^, zeaxanthin concentrations ranged between 0.07 and 8.47 mg·kg^−1^, whereas β-carotene ranged between 0.00 and 0.23 mg·kg^−1^. Notably, the lutein and zeaxanthin concentrations in our RIL population (lutein: 0.04–18.11 mg·kg^−1^; zeaxanthin: 0.07–8.47 mg·kg^−1^) cover and, in the case of lutein, exceed the upper bounds of ranges previously reported for panels of stabilized cultivars [6,24]. This is not a direct comparison but an expected outcome of our experimental design: unlike genome-wide association studies (GWAS) using diverse cultivars, our biparental cross actively recombines alleles from two extreme parents, thereby generating novel genetic combinations and revealing transgressive segregation that is typically fixed or absent in adapted varieties. The coefficient of variation (CV) for carotenoid components in the RIL population exceeded 100% (Table 1), reflecting extensive transgressive segregation. This high CV validates rich genetic diversity and provides a solid foundation for subsequent genetic model dissection. These features may provide useful donor alleles for future breeding within similar genetic backgrounds and offer candidate lines for further marker-assisted improvement. This broader variation is expected, as our biparental population was specifically designed. The observed extensive transgressive segregation for all carotenoid components suggests a complex genetic basis, likely arising from complementary gene action between the parental lines. This is further supported by the high phenotypic variability and continuous, right-skewed distribution, which are hallmarks of quantitative traits controlled by multiple genes. It should be noted that these comparisons are intended only to validate our phenotyping protocol and the b*-carotenoid relationship; they do not imply that the exact ranges observed here will necessarily apply to other genetic backgrounds.

### 3.2. Genetic Model Analysis of Kernel Color and Carotenoid Content in the RIL Population

The major gene plus polygene mixed genetic model is crucial for elucidating the inheritance of quantitative traits. This model has been widely applied in the breeding of rice, wheat, buckwheat, and tobacco [25,26,27,28]. However, in foxtail millet, its use has been largely confined to agronomic traits like plant height and panicle weight [18,29,30]. This study is the first to employ this model for the genetic analysis of quality traits in foxtail millet, namely kernel color and carotenoid content, thereby providing a theoretical basis for quality breeding.

For carotenoids, the inheritance models for lutein, zeaxanthin, β-carotene, and total carotenoid content exhibit a major gene inheritance pattern across both environments. Except for β-carotene, the major gene heritabilities for all other traits exceeded 90%. Comparable findings have been reported in *Tagetes erecta*, where lutein content is also predominantly governed by major genes, with heritability estimates ranging from 60.82% to 86.86% across generations [27]. However, studies in other species reveal different genetic architectures. Wang reported that carotenoid content in wheat is regulated by both major genes and polygenes, with polygenic heritability exceeding that of major genes [16]. In our study, all carotenoid traits could be explained by MX3-AI-A. This similarity in genetic models among correlated traits may stem from gene linkage, as traits sharing biological pathways often exhibit congruent genetic architectures [23]. Consequently, it is plausible that QTLs governing traits related to carotenoid content are situated near similar genetic loci [31]. Nevertheless, it is important to note that the major gene-polygene model has limited resolution for distinguishing tightly linked loci, as it relies on phenotypic segregation rather than molecular markers. Therefore, while our model provides strong evidence for major gene control, it cannot definitively determine whether the different carotenoid components are controlled by the same set of genes or by different but tightly linked loci. Future studies combining fine-mapping with transcriptome analysis would be necessary to dissect the precise genetic architecture and identify the underlying genes. Interestingly, the genetic models for b* value showed environment-dependent variation (MX2-IE-A and 3MG-CEA, respectively). This discrepancy between the stable MX3-AI-A model for carotenoids and the plastic models for b* value exemplifies a core principle: composite traits integrating multiple physiological processes (e.g., b* value = carotenoid pigments + light scattering) are more susceptible to environmental modulation than single-component traits. This occurs because scattering effects from color are sensitive to temperature during grain filling, altering the optical properties of kernels independently of pigment concentration. Consequently, while breeding for high carotenoid content, governed by stable major genes, can be prioritized across environments, improvement of b* value, due to its environmentally plastic genetic architecture, may require specific strategies tailored to target environments.

### 3.3. Prospects for Breeding High-Carotenoid Millet

**Figure 5:**
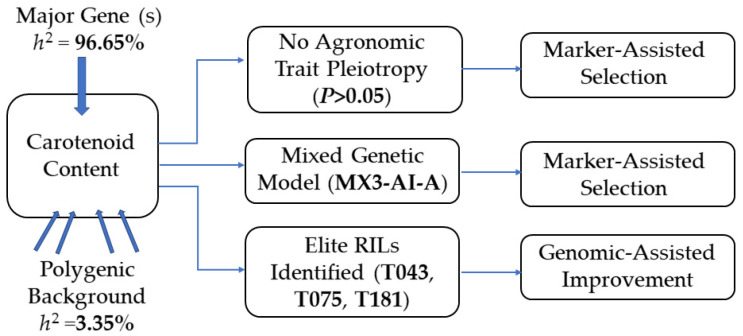
Genetic architecture and breeding pipeline for high-carotenoid foxtail millet, derived from the major-gene plus polygene model. Major-gene heritability (*h*^2^) = 96.65%; polygene heritability = 3.35% (data from Table 4). Carotenoid content shows no significant correlation with yield (*p* > 0.05; data from Figure 3). Solid arrows indicate genetic effects; the dashed arrow denotes the selection pathway. Elite RILs identified in this study are presented in Figure 4.

The genetic insights obtained from the distribution and model analyses are synthesized into a conceptual breeding framework (Figure 5). This diagram illustrates how the key findings logically converge to inform two complementary strategies for the genetic improvement of carotenoid content in foxtail millet. Carotenoids such as lutein, zeaxanthin, and β-carotene can be metabolically converted into vitamin A [32,33,34]. Biofortification of crop quality traits through breeding approaches such as hybridization can effectively enhance vitamin A supply, thereby alleviating micronutrient deficiencies [35,36]. For example, the maize cultivar Pusa Vivek QPM 9 has been developed through hybridization to increase its vitamin A content [37]. Analyzing the genetic patterns of carotenoids in foxtail millet can facilitate breeding efforts. Research in maize has demonstrated that significant epistatic interactions between the key genes *lcyE* and *crtRB1* can regulate the ratio of lutein to zeaxanthin in the endosperm [38]. In this study, carotenoid content was found to be controlled by three major genes exhibiting additive–epistatic effects. Therefore, in genomic selection for breeding, we should not focus solely on the pyramiding of certain genes. Instead, it is crucial to incorporate both additive and epistatic effects between genes to fully exploit the genetic potential. Hybridization can be employed to transfer these additive effects of quantitative traits, enabling the development of foxtail millet varieties with high carotenoid content.

Additionally, consistent with previous research, this study found no correlation between carotenoid content and agronomic traits in foxtail millet. This indicated that breeding for high provitamin A can be pursued concurrently with breeding for high yield. Such an approach allows for the simultaneous improvement of both nutritional quality and grain production. This strategy meets consumer demands while ensuring profitability for farmers. Similar findings have been reported in maize [20]. Based on the favorable traits of the selected elite RILs (Figure 4), we have initiated a practical breeding pipeline. These lines are currently being used as donor parents in marker-assisted backcrossing and hybrid breeding programs, providing a starting point that can accelerate subsequent phenotyping and selection cycles.

## 4. Materials and Methods

### 4.1. Plant Materials and Population Development

A RIL population was developed by crossing Jiugu 25 (JG25) with Jingu 21 (JG21). JG25 (female parent) was bred by the Jilin Academy of Agricultural Sciences, and is characterized by its low carotenoid content, yellow glumes, and purple seedlings. JG21 (male parent), a widely cultivated variety developed by the Institute of Crop Sciences, Shanxi Agricultural University (Shanxi Academy of Agricultural Sciences), which is known for high carotenoid content with green seedlings and golden yellow shiny kernel. The population was advanced to the F_8_ generation using the single-seed descent (SSD) method, ultimately comprising 305 lines. Specifically, from the F_2_ generation onward, one panicle was randomly harvested per line in each generation; all seeds from that panicle were sown together in one row, and one random plant within the row was then chosen to provide the panicle for the next generation. Critically, no selection for carotenoid content, plant vigor, kernel color, or any other trait was practiced throughout this process. This ensured that the final 305 F_8_ lines constitute an unbiased sample of the original segregation spectrum, making the population ideal for genetic analysis.

### 4.2. Field Experiments

Field experiments were conducted in two environments: (1) the South China Breeding Station of Shanxi Agricultural University (Sanya, Hainan; 18.4 °N, 109.5 °E) from December 2023 to March 2024 (denoted as 23HN); (2) the Dongyang Experimental Station of Shanxi Agricultural University (Jinzhong, Shanxi; 37.4 °N, 112.7 °E) from May 2024 to October 2024 (denoted as 24YD). Each environment included three replications. Each plot consisted of six rows (2 m long) with 30 cm between rows and 10 cm between plants. Standard agronomic practices for irrigation, fertilization, and weed control were applied uniformly across all plots. Uniform agronomic management was applied across both environments: a base fertilizer of compound fertilizer (N-P_2_O_2_-K_2_O = 2:1:2) was applied at 140 kg·hm^−2^, and by manual weeding once or twice during the seedling stage. The two sites differed primarily in their climatic and edaphic conditions. The Hainan site (23HN) has a tropical monsoon climate with a mean annual temperature of 25.4 °C and annual precipitation of 1279 mm. In contrast, the Shanxi site (24YD) experiences a temperate continental climate with a mean annual temperature of 9.8 °C and annual precipitation of 450 mm. All other management practices, including the irrigation, fertilization, and weed-control regimes described above, were kept identical between the two environments to ensure comparability.

### 4.3. Phenotypic Evaluation

At physiological maturity, plants from each line were hand-harvested and threshed. The dried grains were dehulled to obtain kernels for subsequent analysis, which were stored at −20 °C.

#### 4.3.1. Kernel Color Measurement

The b* values were measured using a YS3010 grating spectrophotometer (Shenzhen 3nh Technology Co., Ltd., Guangzhou, China). Three technical replicates were performed for each line, and the mean values were used for analysis.

#### 4.3.2. Extraction and Determination of Carotenoids

Carotenoid content was measured in accordance with the method of Li [39] for quantitative analysis. After grinding and sieving through a 60-mesh screen, the samples were saponified at 70 °C for 45 min using a mixture of 95% ethanol, sodium chloride (17.52 g·L^−1^), pyrogallol ethanol solution (63.055 g·L^−1^), ascorbic acid (176 mg·mL^−1^), and potassium hydroxide. The saponified mixture was then subjected to six sequential extractions with n-hexane/ethyl acetate (9:1, *v*/*v*). Combined supernatants were evaporated under nitrogen gas and reconstituted in methyl tert-butyl ether (MTBE) containing 0.1% (*w*/*v*) butylated hydroxytoluene (BHT) for HPLC analysis.

Quantification of lutein, zeaxanthin, and β-carotene was performed using an Agilent 1200 HPLC system (Agilent Technologies, Santa Clara, CA, USA), equipped with a photodiode array detector and a YMC™ carotenoids 30 column (5 μm, 4.5 × 250 mm, Waters Corporation, Wilmington, NC, USA). The column temperature was maintained at 25 °C, and the detection wavelength was set at 450 nm. Mobile phase A consisted of 90% A’ (97% methanol-water solution containing 0.05 M ammonium acetate and 0.1% BHT, *w*/*v*) and 10% B’ (MTBE containing 0.1% BHT, *w*/*v*), while mobile phase B consisted of 90% B’ and 10% A’. Gradient elution was performed at a flow rate of 1 mL·min−1 with the following program: 0–10 min (90–60% A); 10–20 min (60–50% A); 20–25 min (50–10% A); 25–29 min (10% A); 29–29.5 min (10–90% A); 29.5–40 min (90% A). The composition of carotenoids was identified and quantified by comparing the retention times and peak areas of the samples with those of the standards (Sigma, Rödermark, Germany) using the external standard method. Results were expressed as mg·kg^−1^ dry weight (DW). All experiments were performed in triplicate.

#### 4.3.3. Investigation of Agronomic Traits

At maturity, five representative plants from the middle of each row were selected. Eight agronomic traits were investigated according to *Descriptors and Data Standard for Foxtail Millet Germplasm Resources* [40], including plant height (PH), stem length (SL), panicle length (PL), panicle diameter (PD), stem node number (SNN), spike number (SN), spike weight per plant (SWP), and grain weight per plant (GWP).

### 4.4. Data Analysis

Phenotypic data analyses, including descriptive statistics and analysis of variance (ANOVA), were performed using SPSS 26.0 (IBM Corp., Armonk, NY, USA). Frequency distribution histograms were generated using R software (v4.3.1). Correlation analysis was conducted and visualized using Origin 2021 (Origin Lab Corp., Northampton, MA, USA).

Genetic architecture was analyzed through the major gene plus polygene mixed inheritance model implemented in the “SEA v2.0” R package [41]. The best-fit model for each trait was selected based on the smallest Akaike Information Criterion (AIC) value [42]. Candidate models were identified, which were subsequently subjected to goodness-of-fit tests. Finally, first-order and second-order genetic parameters of the optimal genetic model were calculated using this software package [43].

## 5. Conclusions

This study dissected carotenoid inheritance in foxtail millet using 305 RILs and a major-gene-plus-polygene model: three major-gene pairs explain 96.65% of the variance, and the trait is genetically independent of eight yield characters (|r| ≤ 0.11), enabling simultaneous biofortification and yield gain. Three elite F_8_ lines with >23% higher carotenoid and unchanged agronomic performance are already in marker-assisted backcross pipelines. The MX3-AI-A framework and associated germplasm provide breeders with an immediate, low-risk route to release high-yielding, carotenoid-biofortified varieties within one selection cycle.

## Figures and Tables

**Table 1:** Descriptive statistics of carotenoid components and b* value in the parental lines and recombinant inbred line (RIL) population under two environments.

Trait	Env.	Parent		RIL					
		JG 25	JG 21	Mean	Max	Min	CV (100%)	Skewness	Kurtosis
Lutein (mg·kg^−1^)	23HN	0.59	10.81 ****	2.88	17.27	0.06	131.12	1.56	1.32
	24DY	0.54	15.52 ****	3.39	18.11	0.04	132.27	1.51	0.99
Zeaxanthin (mg·kg^−1^)	23HN	0.35	3.46 ****	1.40	6.80	0.07	108.82	1.55	1.32
	24DY	0.35	3.44 ****	1.12	8.47	0.07	137.04	2.42	6.52
β-carotene (mg·kg^−1^)	23HN	0.02	0.10 ***	0.05	0.18	0.00	78.96	0.98	0.55
	24DY	0.01	0.16 ***	0.05	0.23	0.00	89.76	0.96	0.18
Carotenoids (mg·kg^−1^)	23HN	0.95	14.38 ****	4.32	23.69	0.15	122.22	1.53	1.18
	24DY	0.90	19.11 ****	4.56	24.34	0.12	128.86	1.52	1.04
b* value	23HN	19.48	28.21 ***	20.13	32.22	14.83	15.97	1.04	0.76
	24DY	20.10	28.16 ***	23.48	33.91	14.24	15.46	0.40	−0.33

Significance levels are indicated as follows: *** *p* < 0.001, **** *p* < 0.0001.

**Table 2:** Comparison of agronomic traits between selected transgressive segregation families and the population mean under two environments. No significant differences were detected (*p* > 0.05).

Trait	23HN			24DY		
	Extreme Yellowness	Extreme Whiteness	Mean	Extreme Yellowness	Extreme Whiteness	Mean
PH (cm)	54.84 ± 8.81	55.51 ± 12.74	55.54 ± 11.71	102.17 ± 12.12	101.60 ± 9.05	101.50 ± 10.96
PL (cm)	24.86 ± 2.78	24.99 ± 3.32	25.28 ± 3.50	20.89 ± 4.50	19.84 ± 4.27	21.34 ± 3.88
PD (mm)	16.71 ± 4.84	16.98 ± 2.26	16.70 ± 2.36	24.74 ± 3.67	24.29 ± 3.41	24.53 ± 4.30
SWP (g)	10.91 ± 4.07	9.74 ± 3.91	9.33 ± 3.15	18.11 ± 6.82	15.68 ± 2.97	15.76 ± 4.31
GWP (g)	8.97 ± 3.34	8.09 ± 2.93	7.70 ± 2.58	13.78 ± 5.31	12.34 ± 2.49	12.21 ± 3.54

**Table 3:** Akaike information criterion (AIC) values of candidate genetic models for carotenoid content and b* value in the RIL population under two environments.

Model	Env.	AIC Value				
		Lutein	Zeaxanthin	β-Carotene	Carotenoids	b* Value
MX2-A-A	23HN	1419.293	914.835	−1211.720	1535.031	1613.903
	24DY	1590.736	988.844	−1218.261	1682.738	1653.414
MX2-ED-A	23HN	1417.117	905.057	−1216.571	1534.897	1556.636
	24DY	1575.886	984.336	−1114.716	1672.247	1649.259
MX2-IE-A	23HN	1463.223	1077.201	−1183.200	1782.072	1550.358
	24DY	1605.408	1059.933	−1068.500	1787.919	1663.477
3MG-A	23HN	1695.364	796.971	−838.307	1581.257	**1542.646**
	24DY	1818.299	1153.899	−749.837	1627.431	1685.323
3MG-CEA	23HN	1540.914	826.547	−1216.284	1520.576	1558.605
	24DY	1670.595	1153.544	−1127.467	1833.748	**1647.735**
MX3-AI-A	23HN	**1225.443**	**642.684**	**−1220.956**	**1317.842**	1552.948
	24DY	1220.331	**626.471**	**−1223.903**	**1380.898**	1654.710
MX3-A-A	23HN	1233.017	773.929	−1189.387	1378.586	1615.492
	24DY	1285.649	792.149	−1195.669	1447.334	1674.454
MX3-CEA-A	23HN	1330.822	749.295	−1186.804	1504.404	1611.904
	24DY	1788.139	767.532	−1063.491	1905.771	1657.954
4MG-AI	23HN	1241.995	659.448	−1220.951	1461.607	1553.080
	24DY	**1207.397**	899.163	−1212.613	1417.898	1658.439

The bold values represent the minimum AIC values for each trait across different environments.

**Table 4:** First-order and second-order genetic parameters of the optimal genetic model for carotenoid content and b* value in the RIL population under two environments.

Traits	Env.	First-Order Parameters								Second-Order Parameters			
		d (da)	db	dc	iab (i*)	iac	ibc	iabc	[d]	σ^2^_mg_	h^2^_mg_ (%)	σ^2^_pg_	h^2^_pg_ (%)
Lutein	23HN	3.1296	1.6916	2.4283	0.7002	1.4368	−0.0011	−0.8960	−1.3718	13.6046	96.3045	0.0000	0.0000
	24DY	3.6390	2.0858	2.7897	0.7846	1.4885	−0.0647	−1.2538	0.2244	19.1138	96.8203	0.6277	3.1794
Zeaxanthin	23HN	1.6211	1.0478	1.3205	0.0028	0.2755	−0.2978	−0.0164	−2.4230	2.2187	95.9033	0.0947	4.0938
	24DY	1.9575	1.2210	1.6134	0.0665	0.4590	−0.2775	0.3762	−3.6241	2.2526	96.1372	0.0905	3.8628
β-carotene	23HN	0.0208	0.0164	0.0192	0.0039	0.0067	0.0023	−0.0033	−0.0095	0.0012	80.6935	0.0003	19.2824
	24DY	0.0390	0.0167	0.0241	0.0017	0.0091	0.0073	−0.0052	0.0015	0.0023	94.7691	0.0001	5.2307
carotenoid	23HN	4.5248	1.1623	3.5555	−0.3323	2.0609	−1.3015	−2.4462	−0.0834	26.8537	96.6448	0.9323	3.3552
	24DY	4.5113	2.8266	3.6099	0.8343	1.6175	−0.0671	−1.6458	−0.1961	32.9328	96.6526	1.1406	3.3474
b* value	23HN				2.8742				4.3633	6.1085	59.7983	2.7860	27.2734
	24DY	1.7121	1.7121	1.7121						9.5873	75.6566		

da: Additive effect of the first major gene; db: Additive effect of the second major gene; dc: Additive effect of the third major gene; i: Epistatic effect value; [d]: Additive effect of polygene; σ^2^_pg_: Polygene variance; σ^2^_mg_: Major gene variance; h^2^_mg_ (%): Heritability of major gene; h^2^_pg_ (%): Heritability of polygene.

## Data Availability

The original contributions presented in this study are included in the article. Further inquiries can be directed to the corresponding authors.

## Appendix A

**Table A1:** Goodness-of-fit tests for candidate genetic models of carotenoid content and b* value in the RIL population under two environments.

Trait	Env.	Model	Generation	U_1_^2^	U_2_^2^	U_3_^2^	_n_W^2^	D_n_
Lutien	23HN	MX3-AI-A	P_1_	8.0471 (0.0046) *	3.7263 (0.0536)	10.6617 (0.0011) *	0.8929 (0.0044) *	0.9495 (0.0003) *
		MX3-AI-A	P_2_	8.2321 (0.0041) *	3.7393 (0.0531)	11.4064 (0.0007) *	0.9346 (0.0035) *	0.9770 (0.0000) *
		MX3-AI-A	RIL	0.0428 (0.8361)	0.4534 (0.5007)	3.5793 (0.0585)	0.4336 (0.0609)	0.1173 (0.0005) *
		MX3-A-A	P_1_	0.0899 (0.7643)	0.6690 (0.4134)	4.4542 (0.0348) *	0.1058 (0.5667)	0.3333 (0.7778)
		MX3-A-A	P_2_	0.0022 (0.9627)	0.1632 (0.6862)	3.2307 (0.0723)	0.1445 (0.4088)	0.4254 (0.5246)
		MX3-A-A	RIL	0.6392 (0.4240)	1.1386 (0.2860)	1.3729 (0.2413)	0.6415 (0.0178) *	0.1123 (0.0010) *
	24DY	4MG-AI	P_1_	6.3108 (0.0120) *	3.6026 (0.0577)	4.5676 (0.0326) *	0.7723 (0.0085) *	0.9160 (0.0012) *
		4MG-AI	P_2_	0.5198 (0.4709)	1.2059 (0.2721)	2.5608 (0.1095)	0.2843 (0.1565)	0.6133 (0.1261)
		4MG-AI	RIL	0.0484 (0.8260)	0.0223 (0.8812)	0.0644 (0.7997)	0.0725 (0.7446)	0.0561 (0.2925)
		MX3-AI-A	P_1_	0.0010 (0.9742)	0.0053 (0.9422)	0.1725 (0.6779)	0.0342 (0.9604)	0.2268 (0.9896)
		MX3-AI-A	P_2_	0.0010 (0.9742)	0.0053 (0.9422)	0.1725 (0.6779)	0.0342 (0.9604)	0.2268 (0.9896)
		MX3-AI-A	RIL	0.0063 (0.9369)	0.2630 (0.6081)	3.0449 (0.0810)	0.2534 (0.1898)	0.0990 (0.0051) *
Zeaxanthin	23HN	MX3-AI-A	P_1_	0.0000 (1.0000)	0.0108 (0.9172)	0.1729 (0.6776)	0.0341 (0.9607)	0.2230 (0.9914)
		MX3-AI-A	P_2_	0.0000 (1.0000)	0.0108 (0.9172)	0.1729 (0.6776)	0.0341 (0.9607)	0.2230 (0.9914)
		MX3-AI-A	RIL	0.1671 (0.6827)	0.3947 (0.5299)	0.8643 (0.3525)	0.2038 (0.2624)	0.0559 (0.3009)
		4MG-AI	P_1_	0.0924 (0.7611)	0.5810 (0.4459)	3.5026 (0.0613)	0.2337 (0.2151)	0.5321 (0.2627)
		4MG-AI	P_2_	2.1733 (0.1404)	2.4334 (0.1188)	0.2811 (0.5960)	0.4115 (0.0699)	0.7306 (0.0391)
		4MG-AI	RIL	0.0236 (0.8780)	0.0218 (0.8827)	1.4031 (0.2362)	0.0840 (0.6809)	0.0490 (0.4622)
	24DY	MX3-AI-A	P_1_	0.0000 (1.0000)	0.0108 (0.9172)	0.1729 (0.6776)	0.0341 (0.9607)	0.2230 (0.9914)
		MX3-AI-A	P_2_	0.0000 (1.0000)	0.0108 (0.9172)	0.1729 (0.6776)	0.0341 (0.9607)	0.2230 (0.9914)
		MX3-AI-A	RIL	0.1034 (0.7477)	1.0163 (0.3134)	7.7664 (0.0053) *	0.7826 (0.0081) *	0.1306 (0.0001) *
		MX3-CEA-A	P_1_	0.0003 (0.9863)	0.0145 (0.9041)	0.1728 (0.6777)	0.0341 (0.9606)	0.2251 (0.9904)
		MX3-CEA-A	P_2_	0.0003 (0.9863)	0.0145 (0.9041)	0.1728 (0.6777)	0.0341 (0.9606)	0.2251 (0.9904)
		MX3-CEA-A	RIL	2.8978 (0.0887)	8.0862 (0.0045) *	22.8628 (0.0000) *	2.6222 (0.0000) *	0.1785 (0.0000) *
β-carotene	23HN	MX3-AI-A	P_1_	0.0424 (0.8368)	0.0145 (0.9041)	1.6378 (0.2006)	0.0617 (0.8066)	0.3027 (0.8791)
		MX3-AI-A	P_2_	0.1708 (0.6794)	0.7051 (0.4011)	3.0906 (0.0787)	0.1846 (0.3006)	0.5022 (0.3282)
		MX3-AI-A	RIL	0.0284 (0.8662)	0.0129 (0.9094)	1.2266 (0.2681)	0.2863 (0.1546)	0.0863 (0.0223) *
		4MG-AI	P_1_	8.8640 (0.0029) *	3.7497 (0.0528)	14.3279 (0.0002) *	0.9875 (0.0026) *	0.9953 (0.0000) *
		4MG-AI	P_2_	0.0712 (0.7895)	0.5344 (0.4648)	3.5734 (0.0587)	0.2517 (0.1919)	0.5413 (0.2440)
		4MG-AI	RIL	0.0633 (0.8014)	0.0480 (0.8267)	0.0097 (0.9216)	0.1304 (0.4593)	0.0483 (0.4805)
	24DY	MX3-AI-A	P_1_	0.0013 (0.9717)	0.0331 (0.8557)	0.7483 (0.3870)	0.0448 (0.9072)	0.2622 (0.9582)
		MX3-AI-A	P_2_	0.0035 (0.9530)	0.0000 (0.9977)	0.0575 (0.8105)	0.0716 (0.7495)	0.3419 (0.7549)
		MX3-AI-A	RIL	0.0409 (0.8398)	0.0292 (0.8644)	2.1495 (0.1426)	0.3844 (0.0830)	0.1020 (0.0035) *
		MX2-A-A	P_1_	0.0000 (0.9979)	0.0458 (0.8306)	0.7496 (0.3866)	0.0447 (0.9076)	0.2589 (0.9623)
		MX2-A-A	P_2_	0.0105 (0.9182)	0.0021 (0.9638)	0.0466 (0.8291)	0.0727 (0.7434)	0.3506 (0.7312)
		MX2-A-A	RIL	0.0223 (0.8812)	0.3093 (0.5781)	2.7077 (0.0999)	0.5014 (0.0402) *	0.1079 (0.0017) *
carotenoids	23HN	MX3-AI-A	P_1_	0.0000 (0.9947)	0.2231 (0.6367)	3.4743 (0.0623)	0.1681 (0.3397)	0.4327 (0.5049)
		MX3-AI-A	P_2_	0.0000 (0.9974)	0.1048 (0.7461)	1.7094 (0.1911)	0.0587 (0.8244)	0.2912 (0.9073)
		MX3-AI-A	RIL	0.0075 (0.9311)	0.0949 (0.7580)	2.4560 (0.1171)	0.2822 (0.1586)	0.0828 (0.0317)
		MX3-A-A	P_1_	0.0000 (0.9993)	0.1373 (0.7109)	2.1876 (0.1391)	0.0799 (0.7031)	0.3283 (0.7973)
		MX3-A-A	P_2_	0.0000 (0.9998)	0.2341 (0.6285)	3.7497 (0.0528)	0.0833 (0.6845)	0.3333 (0.7778)
		MX3-A-A	RIL	0.0388 (0.8439)	0.4389 (0.5077)	3.5621 (0.0591)	0.5081 (0.0386) *	0.0926 (0.0112) *
	24DY	MX3-AI-A	P_1_	0.0000 (0.9973)	0.1047 (0.7463)	1.7094 (0.1911)	0.0587 (0.8244)	0.2912 (0.9073)
		MX3-AI-A	P_2_	0.0000 (0.9945)	0.2234 (0.6364)	3.4743 (0.0623)	0.1681 (0.3397)	0.4327 (0.5047)
		MX3-AI-A	RIL	0.0327 (0.8566)	0.1084 (0.7420)	4.0677 (0.0437)	0.3253 (0.1209)	0.1075 (0.0017) *
		4MG-AI	P_1_	8.1709 (0.0043) *	3.7375 (0.0532)	11.141 (0.0008) *	0.9308 (0.0036) *	0.9763 (0.0000) *
		4MG-AI	P_2_	0.4450 (0.5047)	1.1198 (0.2900)	2.7200 (0.0991)	0.2870 (0.1539)	0.6111 (0.1289)
		4MG-AI	RIL	0.0599 (0.8066)	0.0807 (0.7763)	0.0354 (0.8507)	0.0898 (0.6495)	0.0622 (0.1889)
b* value	23HN	3MG-A	P_1_	4.6020 (0.0319) *	5.6700 (0.0173) *	1.4794 (0.2239)	0.5129 (0.0375) *	0.7544 (0.0296) *
		3MG-A	P_2_	3.4733 (0.0624)	2.7365 (0.0981)	0.3613 (0.5478)	0.4035 (0.0735)	0.7060 (0.0508)
		3MG-A	RIL	0.2361 (0.6271)	0.2004 (0.6544)	0.0083 (0.9276)	0.1729 (0.3277)	0.0493 (0.4514)
		MX2-IE-A	P_1_	1.0927 (0.2959)	0.8689 (0.3512)	0.1023 (0.7491)	0.1440 (0.4104)	0.4639 (0.4223)
		MX2-IE-A	P_2_	0.7127 (0.3985)	0.7633 (0.3823)	0.0506 (0.8220)	0.1153 (0.5221)	0.4306 (0.5105)
		MX2-IE-A	RIL	0.0776 (0.7806)	0.2284 (0.6327)	0.6937 (0.4049)	0.1412 (0.4200)	0.0543 (0.3317)
	24DY	3MG-CEA	P_1_	1.8700 (0.1715)	1.6343 (0.2011)	0.0333 (0.8552)	0.2193 (0.2362)	0.4898 (0.3576)
		3MG-CEA	P_2_	1.1770 (0.2780)	0.6301 (0.4273)	1.0540 (0.3046)	0.2526 (0.1908)	0.5672 (0.1958)
		3MG-CEA	RIL	0.0128 (0.9100)	0.0005 (0.9822)	0.2778 (0.5982)	0.0767 (0.7208)	0.0421 (0.6512)
		MX2-ED-A	P_1_	0.2601 (0.6100)	0.0123 (0.9117)	2.3464 (0.1256)	0.1140 (0.5280)	0.4204 (0.5384)
		MX2-ED-A	P_2_	0.2230 (0.6367)	0.5552 (0.4562)	1.3254 (0.2496)	0.1133 (0.5310)	0.4565 (0.4414)
		MX2-ED-A	RIL	0.0055 (0.9406)	0.0211 (0.8845)	0.0857 (0.7697)	0.0475 (0.8916)	0.0277 (0.9731)

* means significant difference (<0.05). Probability values are shown in brackets.

**Table A2:** Number of Families and Carotenoid Content in Different Groups.

Cluster	Carotenoid Content (mg·kg^−1^)	Number
Cluster I	1.53	230
Cluster II	10.85	29
Cluster III	16.97	46
